# Is yogurt intake associated with periodontitis due to calcium?

**DOI:** 10.1371/journal.pone.0187258

**Published:** 2017-10-30

**Authors:** Hye-Sung Kim, Young-Youn Kim, Jeong-Kyu Oh, Kwang-Hak Bae

**Affiliations:** Oral Health Science Research Center, Apple tree Dental Hospital, Goyang-si, Gyounggi-do, Korea; University of Insubria, ITALY

## Abstract

The purpose of this study is to determine whether the lower intakes of yogurt, milk, and calcium are associated with periodontitis in a nationally representative sample of Korean adults. This study comprised 6,150 adults 19 or more years old who took both periodontal examination and nutrition survey. The frequency of yogurt and milk intake was examined with a food frequency questionnaire. The amount of calcium intake was calculated with dietary intakes data gained from complete one-day 24-hour recall interviews. Periodontitis was assessed using the Community Periodontal Index (CPI). Multivariate logistic regression analyses were performed for the whole sample and subgroups with the strata of age, gender, or smoking, in a complex sampling design. Less intake of yogurt was significantly associated with periodontitis (odds ratio [OR] 0.82, 95% confidential interval [CI] 0.70–0.97), but neither less intake of milk (OR 1.04, 95% CI 0.89–1.20) nor lower intake of calcium (OR 1.04, 95% CI 0.89–1.21) was significantly associated with periodontitis. In the subgroup analysis, no difference in the association of yogurt intake with periodontitis was found according to the strata of age, gender, and smoking. In conclusion, periodonitis was significantly associated with the less intake of yogurt among the Korean adults, but the calcium contained in yogurt is not likely to cause it.

## Introduction

Periodontitis is a chronic inflammatory disease that leads to destruction of the connective tissue and alveolar bone around teeth [1]. Due to its high prevalence in adults, it is a major cause of teeth extraction in adults and represents one of the important public health problems that increase the burden of chronic diseases in many countries [2, 3]. In Korea, the prevalence of periodontitis is about 30% among adults in recent years [4].

Several factors were suggested as risk factors of periodontitis such as smoking and diabetes [5,6]. Diet and lower intake of various nutrients were also suggested as potential risk factors for periodontitis [7, 8].

Dairy foods have various kinds of nutrients including calcium, branched chain amino acids, conjugated linoleic acids, protein, vitamin D, and medium-chain fatty acids [9]. In 2006, Al-Zahrani [10] proposed a novel hypothesis that dairy food intake might be inversely associated with periodontitis. The fact that dairy foods have a lot of calcium has been suggested as the reason. This suggestion seems reasonable because lower intake of calcium has been found to be significantly associated with increased prevalence of periodontitis [11]. But, Shimazaki [12] reported a result refuting Al-Zahrani’s hypothesis and also provided potential explanation for it. In Hisayama study, the association of periodontitis with each type of dairy foods was examined. Among various types of dairy foods, only intake of lactic acid foods such as yogurt was associated with periodontitis. Although the amount of calcium in milk is the same as that in yogurt, the intake of milk does not have a significant effect on periodontitis. Yogurt has similar nutrients to milk, but it is characterized by a lot of lactic acid bacteria as a fermented food. Hence, the suggested reason is that lactic acid foods contain many live probiotics that have good effects on periodontitis [13].

A recent longitudinal study in older Danish adults proposed another hypothesis suggesting that dairy calcium from milk and fermented products may protect against periodontis [14]. Calcium has emerged as an important cause for the association between intake of dairy foods and periodontitis again. But, given some recent studies on the association between probiotics and periodontitis [15,16], probiotics in fermented dairy foods could not be overlooked.

In order to test these conflicting hypotheses, it is necessary to directly investigate the association of yogurt, milk and calcium intake with the prevalence of periodontitis. We hypothesized that if intake of dairy foods is related to periodontitis due to probiotics but not to calcium, only yogurt intake would be associated with periodontitis, and milk or calcium intake would not. Therefore, we aimed to determine whether the lower intake of yogurt, milk, and calcium are associated with periodontitis from a nationally representative sample of Korean adults.

## Materials and methods

### Study design and subject selection

The data used in this study are a subset of the fourth Korea National Health and Nutrition Examination Survey (KNHANES) conducted in 2009 by Korea Centers for Disease Control and Prevention (KCDC) [17]. The sampling protocol for the KNHANES was designed to involve a complex, stratified, multistage, probability-cluster survey of a representative sample of the non-institutionalized civilian population in Korea. The survey was performed by the Korean Ministry of Health. The target population of the survey was all non-institutionalized civilian Korean individuals aged 1 year or older. The survey employed stratified multistage probability sampling units based on geographic area, gender, and age, which were determined based on the household registries of the 2005 National Census Registry, the most recent 5-year national census in Korea. Using the 2005 census data, 200 primary sampling units (PSU) were selected annually across Korea. The details for sampling are described in the previous study for the association between manganese and periodontal status using this data and the fourth KNHANES reports [18, 19].

The sample set for 2009 KNHANES included 4,600 households and 10,533 participants. Of these participants, 7,095 individuals aged 19 years and older had a periodontal examination. Among them, 6,150 individuals who took a one-day 24-hour recall interview and responded to food frequency questionnaire were included in the final sample for this study.

### Clinical variables

#### Periodontitis

The World Health Organization (WHO) community periodontal index (CPI) was used to assess periodontitis [20]. Periodontitis was defined as a CPI greater than or equal to ‘code 3’, which indicates that at least one site had a probing pocket depth > 3.5 mm (code 4 > 5.5 mm). Index tooth numbers were 11, 16, 17, 26, 27, 31, 36, 37, 46 and 47.

A CPI probe (Osung MND CO. Ltd., Seoul, Korea) that met the WHO guidelines was used [20]. The mouth was divided into sextants, and approximately 20 g force was used when probing. In the 2009 KNHANES, 27 trained dentists examined the periodontal status of the participants. The inter-examiner mean of Kappa value was 0.77 (0.53 to 0.94) [21].

#### Calcium intake levels and the frequency of yogurt & milk intake

Nutrition surveys for gathering the data of calcium intake levels and the frequency of yogurt & milk intake were conducted by trained nutritionists at participant’s home. Each survey team was composed of two trained nutritionists. The quality control on the nutritional survey was conducted by the Center for Nutrition Policy and Promotion at Korea Health Industry Development Institute [18,22].

The daily calcium intake was converted from food intake data gained using a complete individual one-day 24-hour recall interview. Calcium intake was calculated by multiplying the weight for each food item which a participant reported to take in by calcium concentration data for the corresponding food code in Korean food composition table [23]. The frequency of yogurt and milk intake was divided into two groups by once a week. The calcium intake levels of participants were also divided into two groups by a median value.

#### Covariates

Socio-demographic variables comprised gender, age, household income and education level. Household income was the family income adjusted for the number of family members and converted quartiles. Education level was defined as the highest diploma the participant had received.

Oral health behaviors included daily frequency of toothbrushing and usage of dental floss or an interdental brush. For smoking status, participants were divided into three groups depending on the status: non-smokers (those who had never smoked or had smoked fewer than 100 cigarettes in their lifetime); current smokers (those who currently smoked and had smoked 100 cigarettes or more in their lifetime); and past smokers (those who had smoked in the past but were not current smokers). Systemic conditions included diabetes and obesity.

### Statistical analysis

The complex sampling design of the survey was considered, and individual weighted factors were used when obtaining variances. Multivariate logistic regression analyses were applied to examine the associations of the frequency of yogurt & milk intake and calcium intake level with periodontitis. The odds ratios (ORs) of yogurt, milk, and calcium intake for periodontitis were adjusted for the above mentioned covariates in the multivariate logistic regression models. In order to find out effect modifiers, subgroup analyses were performed to gain stratified estimates according to gender, age, and current smoking status. Statistical analyses were performed using SPSS statistical software version 19.0 (SPSS, Chicago, IL).

## Results

The prevalence of periodontitis defined as a CPI code ≥ 3 was 30.8% in adults over the age 19 years (n = 6,150). In total participants, mean age (standard deviation) was 48.68 (16.20), female-to-male ratio was 1.44. The total participants consisted of 33% of the 19–39 age group, 54.7% of the 40–69 age group, and 12.3% of the 70 and over age group. Table 1 lists characteristics of participants categorized by periodontitis, including socio-demographic characteristics, oral and systemic health status, and oral and general health behaviors.

**Table 1:** Univariate comparisons of characteristics in participants with and without periodontitis.

Characteristics	No periodontitis		Periodontitis	
	n	Weighted % (95% CI)	n	Weighted % (95% CI)
Socio-demographic variables				
Age (n = 6150)	40.52 (39.72–41.33)^a^		51.57 (50.51–52.63)^a^	
Gender (n = 6150)				
Male	1515	63.2(60.5–65.8)	1008	36.8(34.2–39.5)
Female	2653	75.1 (72.9–77.2)	974	24.9(22.8–27.1)
Highest diploma (n = 6118)				
Primary school	951	53.5(49.5–57.3)	684	46.5(42.7–50.5)
Middle school	417	58.9(53.8–63.7)	289	41.1(36.3–46.2)
High school	1564	73.2(70.4–75.8)	614	26.8(24.2–29.6)
≥ University or College	1215	76.1(73.4–78.6)	384	23.9(21.4–26.6)
Household income^b^ (n = 6089)				
< 25%	755	62.1(58.0–65.9)	479	37.9(34.1–42.0)
25–50%	929	66.7(63.6–69.5)	502	33.3(30.5–36.4)
50–75%	1157	68.4(64.9–71.7)	539	31.6(28.3–35.1)
> 75%	1282	74.6(72.0–77.1)	446	25.4(22.9–28.0)
Systemic health status				
Diabetes (n = 5751)				
Normal	3006	74.0(71.9–75.9)	1142	26.0(24.1–28.1)
Impaired fasting glucose	612	58.0(54.0–61.9)	442	42.0(38.1–46.0)
Diabetes mellitus	283	49.3(44.4–54.3)	266	50.7(45.7–55.6)
BMI^c^ (n = 6106)				
Underweight	200	78.4(72.2–83.6)	63	21.6(16.4–27.8)
Normal	2672	70.8(68.6–73.0)	1216	29.2(27.0–31.4)
Obese	1257	64.0(60.9–67.0)	698	36.0(33.0–39.1)
Oral health behaviors				
Daily frequency of toothbrushing (n = 6139)				
Once or less	484	62.5(58.4–66.4)	310	37.5(33.6–41.6)
Twice	1613	66.4(63.5–69.2)	871	33.6(30.8–36.5)
Three times or more	2062	72.9(70.6–75.2)	799	27.1(24.8–29.4)
Use of floss or interdental brush (n = 6150)				
No	3101	68.2(84.8–90.0)	1572	31.8(10.0–15.2)
Yes	1067	72.0(69.1–74.8)	410	28.0(25.2–30.9)
General health behavior				
Smoking status (n = 6115)				
Non-smoker	2698	75.2(73.0–77.4)	999	24.8(22.6–27.0)
Past smoker	686	62.3(58.7–65.8)	462	37.7(34.2–41.8)
Current smoker	763	61.8(58.2–65.4)	507	38.2(34.6–41.8)
Diet				
Frequency of yogurt intake (n = 6150)				
Under once a week	2694	64.8(62.5–67.1)	1505	35.2(32.9–37.5)
Once a week or more	1474	77.1(74.6–79.3)	477	22.9(20.7–25.4)
Frequency of milk intake (n = 6150)				
Under once a week	1725	63.4(60.6–66.1)	1010	36.6(33.9–39.4)
Once a week or more	2443	72.9(70.7–75.1)	972	27.1(24.9–29.3)
Calcium intake (n = 6149)				
Less than median	2102	69.8(67.2–72.2)	972	30.2(27.8–32.8)
Median or more	2065	68.6(66.1–71.0)	1010	31.4(29.0–33.9)

CPI, Community Periodontal Index; 95% CI, 95% confidence interval.

^a^Weighted mean and 95% confidence interval.

^b^Household income is the monthly average family equivalent income.

(=monthly average household income/√[the number of household members]).

^c^Underweight, <18.5 kg/m^2^; Normal, 18.5–24.9 kg/m^2^; Obese, ≥25 kg/m^2^.

In Table 2, the association between the less yogurt intake and periodontitis was shown in the multivariate logistic regression model, but less milk intake and lower calcium intake were not associated with periodontitis. Less intake of yogurt (under once a week) was significantly associated with periodontitis (Odds ratio 0.82; 95% confidential interval 0.70–0.97).

**Table 2:** Adjusted odds ratios and 95% confidence intervals of yogurt, milk, and calcium intakes for periodontitis.

Explanatory variables	Adjusted odds ratio	95% confidence interval
Yogurt intake		
Under once a week	1	-
Once a week or more	0.822	0.699–0.967
Milk intake		
Under once a week	1	-
Once a week or more	1.035	0.891–1.201
Calcium intake		
Less than median	1	-
Median or more	1.038	0.890–1.209

The odds ratios were adjusted for age, gender, socioeconomic status (education level & household income), health behaviors (smoking, daily frequency of toothbrushing, and usage of dental floss or an interdental brush), and systemic conditions (Diabetes & obesity)

Table 3 shows the results of subgroup analyses. No difference in the association of yogurt intake with periodontitis was found according to the strata of age, gender, and smoking.

**Table 3:** Adjusted odds ratios (OR) and 95% confidence intervals (CI) of yogurt, milk, and calcium intakes for periodontitis in subgroups according to the strata of age, gender, or smoking.

Subgroups	Yogurt	Milk	Calcium
	OR (95% CI)	OR (95% CI)	OR (95% CI)
Age			
19–39	0.776 (0.574–1.049)	1.035 (0.744–1.440)	0.954 (0.696–1.309)
40 or more	0.886 (0.727–1.080)	1.084 (0.915–1.285)	1.036 (0.872–1.231)
Gender			
Men	0.827 (0.665–1.028)	0.925 (0.751–1.139)	1.099 (0.890–1.356)
Women	0.843 (0.675–1.053)	1.222 (0.988–1.510)	0.988 (0.807–1.210)
Current smoker			
No	0.824 (0.682–0.996)	0.985 (0.821–1.182)	1.150 (0.966–1.368)
Yes	0.748 (0.550–1.017)	1.126 (0.812–1.561)	1.073 (0.781–1.476)

The odds ratios were adjusted for age, gender, socioeconomic status (education level & household income), health behaviors (smoking, daily frequency of toothbrushing, and usage of dental floss or an interdental brush), and systemic conditions (Diabetes & obesity).

In subgroup, each effect modifier was excluded from its multivariate model except age.

## Discussion

This study investigates whether lower level of calcium intake and less intake of yogurt or milk were associated with poor periodontal health in Korean adults 19 or more years old after adjusting for socio-demographic variables, oral and general health behaviors, and oral and systemic health statuses.

In this study, less intake of yogurt was significantly associated with periodontitis, but neither less intake of milk nor lower intake of calcium is significantly associated with periodontitis. This finding is consistent with Hisayama study’s result [12] suggesting that the intake of lactic acid foods with probiotics was associated significantly with periodontitis, but that milk intake was not. Several studies reported that probiotics application was likely to have beneficial effects on periodontal disease [16,17,24], and it is suggested that probiotics produce metabolic substance such as reuterin which has the activity inhibiting the growth of periodontopathic bacteria [16]. However, there are few studies on the effect of probiotics of yogurt on periodontitis.

The authors of Hisayama study inferred that calcium intake from dairy products might not have had a major impact on periodontitis, but they did not prove it directly. In this study, it is directly proved that calcium intake may not be associated with periodontitis. Contra the results of this study, a Danish study [14] reported that milk as well as yogurt was associated with periodontitis. But, it did not provide any reasonable explanation for the result that not cheese or non-dairy calcium but milk was associated with periodontitis.

In the subgroup analysis, the association of yogurt intake with periodontitis was not different according to the strata of age, gender, and smoking. Neither milk nor calcium intake was associated with periodontitis in all subgroups, either. We could not find any effect modifications in this analysis.

There are several limitations in this study. First of all, in KNHANES data, the intake level of calcium can be only supposed by the Korean dietary reference intakes (KDRIs) [25]. Therefore, the criteria assessing the internal calcium status included the amounts of intake calculated from dietary survey data but not from biochemical indicators. Even though several countries adopt a 24-hour recall to yield the amount of nutrition intakes [26], it could not perfectly represent habitual calcium intake of individuals due to the shortness of survey days (a one-day 24-hour recall in Korea) and the dependence of the memory of individuals. Thus, using biochemical values would reflect the calcium status more accurately. However, the KDRIs were established as based on biochemical markers, which is one of the biochemical indicators for the calcium level [25].

Another limitation of this study in terms of dental examination is that periodontitis was assessed using the CPI. Although the CPI is an easy way to assess the prevalence of periodontitis in population surveys and epidemiological studies, it may overestimate or underestimate periodontal status due to the use of representative teeth and pseudo-pockets [27]. Also, the type of periodontitis such as chronic or aggressive could not be divided.

The third limitation of this study is that we could not consider the systemic conditions such as gastrointestinal disease and malabsorption, which may affect the intake of dairy foods.

Finally, it is not possible to determine the direction of causal-relationship between yogurt intake and periodontitis since this study is cross-sectional.

Nevertheless, we directly investigated the association of yogurt, milk and calcium intake with the prevalence of periodontitis after adjusting for various potential confounders including sociodemographic variables, oral and general health behavioural factors and systemic health statuses. In addition, this study was carried out comprehensively with a representative Korean population sample.

In conclusion, periodontitis was significantly associated with less intake of yogurt among the Korean adults, but the calcium contained in yogurt is not likely to cause it. Further clinical studies are needed to elucidate the effect of yogurt intake on periodontitis.
